# Synthesis and bioevaluation of thienopyrimidines bearing a pyrazoline unit as selective PI3Kα inhibitors†

**DOI:** 10.1039/c9ra06192d

**Published:** 2019-09-18

**Authors:** Luogen Lai, Qinqin Wang, Binliang Zhang, Zhen Xiao, Zunhua Yang, Qi Yang, Zixin Luo, Wufu Zhu, Shan Xu

**Affiliations:** Jiangxi Provincial Key Laboratory of Drug Design and Evaluation, School of Pharmacy, Jiangxi Science & Technology Normal University Nanchang 330013 China zhuwufu-1122@163.com shanxu9891@126.com +86 791 8380-2393 +86 791 8380-2393; College of Pharmacy, Jiangxi University of Traditional Chinese Medicine Nanchang 330004 China

## Abstract

A series of thienopyrimidines containing a pyrazoline unit (4a–d, 7a–d and 13a–l) were designed and synthesized. The target compounds were evaluated for antiproliferative activity against A549, HepG2 and MCF-7 cancer cell lines. Among the twenty target compounds, most of them exhibited excellent antiproliferative activity against one or several cancer cell lines. Compound 13f showed the best activity against A549, MCF-7 and HepG2 cancer cell lines, with IC_50_ values of 2.84 ± 0.09 μM, 2.88 ± 0.43 μM and 2.08 ± 0.36 μM, respectively. Four selected compounds (13c, 13f, 13g and 13h) were further evaluated for their inhibitory activity against the PI3Kα/mTOR protein kinase. Moreover, time-dependent and dose-dependent experiments, AO fluorescence staining, Annexin V-FITC/PI staining and docking studies were carried out in this study. The results indicated that compound 13f may be a potential selective PI3Kα inhibitor.

## Abstract 2

A series of thienopyrimidines containing a pyrazoline unit (4a–d, 7a–d and 13a–l) were designed and synthesized. The compound 13f showed the best activity with the IC_50_ of 0.92 μM against PI3Kα.

## Graphical abstract

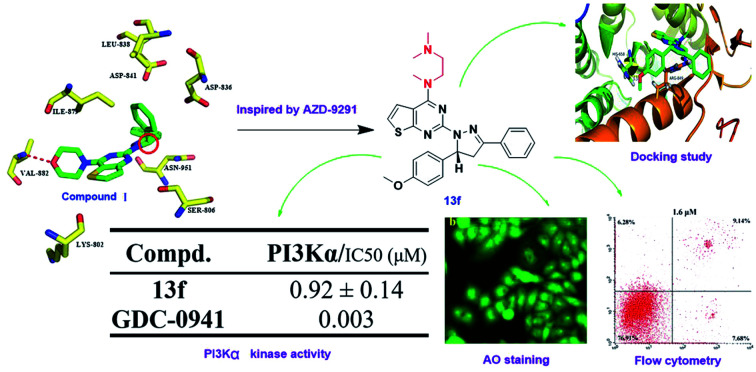

## 1. Introduction

Phosphatidylinositol 3-kinases (PI3Ks) are a family of enzymes involved in cellular functions such as cell growth, proliferation, differentiation, migration, mobility and apoptosis, which in turn are involved in cancer.^1,2^ PI3Ks are currently divided into four different classes: Class I, Class II, Class III, and Class IV.^3^ The Class I family consists of three isoforms, α, β, and δ.^4^ Among the different PI3K subfamily proteins, PI3Kα is the most important isoform in cell proliferation in response to growth factor-tyrosine kinase pathway activation.^5,6^ There are currently more than ten PI3Kα inhibitors in clinical trials, including GDC-0941 and PI-103.^7,8^ Many research groups are attempting to develop some more PI3Kα inhibitors.

**Fig. 1:**
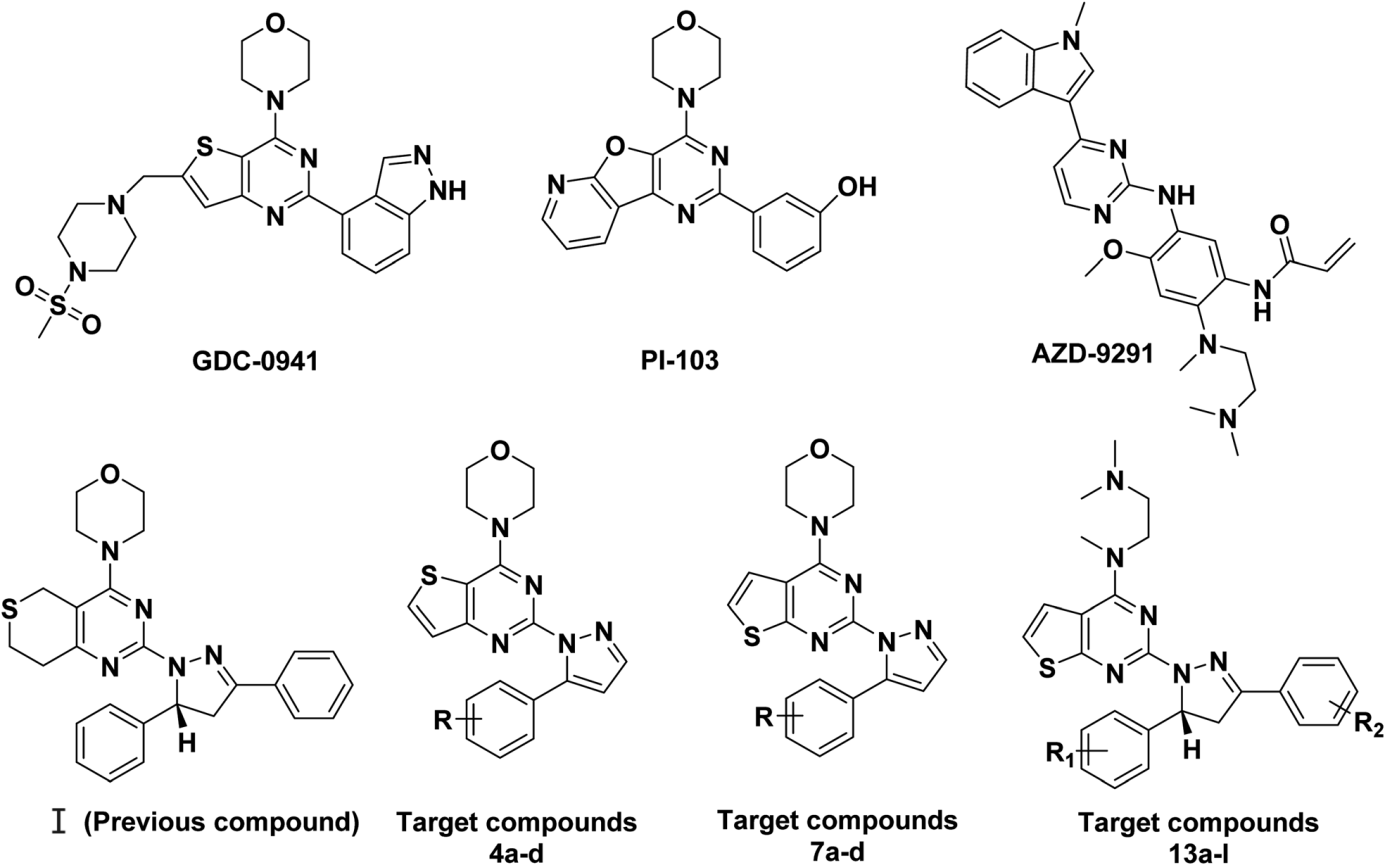
Structures of representative compounds and target compounds.

In our previous research, a series of thianopyrimidine derivatives (compound I, Fig. 1) were designed and synthesized as PI3K inhibitors. Some of them showed moderate to excellent antiproliferative activity. The structure-activity relationships (SARs) and docking studies exhibited that chiral carbon atoms may affect antiproliferative activity *in vitro*.^9^

**Fig. 2:**
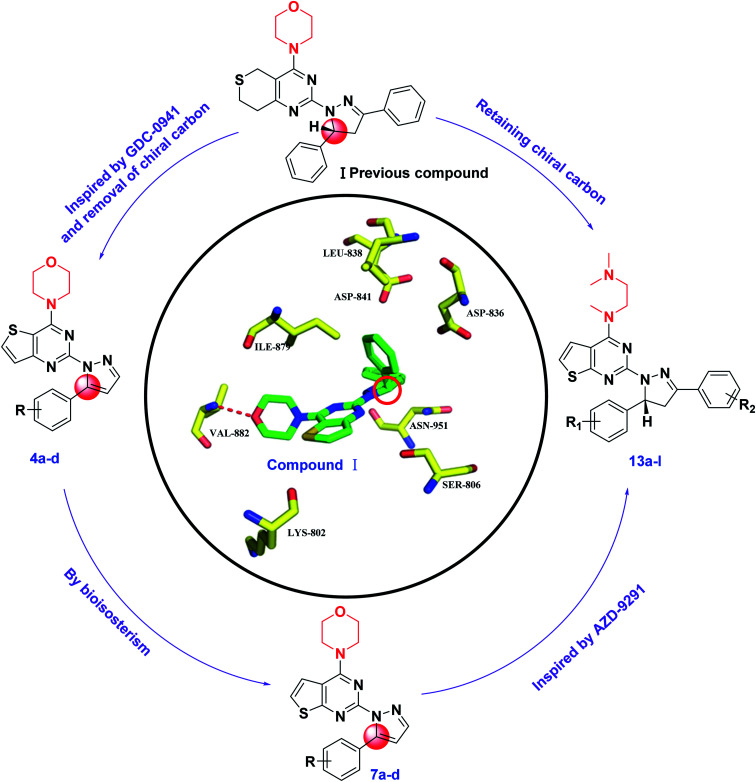
Structures and design strategy for target compounds 4a–d, 7a–d and 13a–l.

To further confirm the effect of the chiral carbon atoms to the activity of the target compounds, we replaced the chiral carbon atoms of the pyrazolines by C=C double bond, and at the same time, inspired by GDC-0941, we replaced the 7,8-dihydro-5*H*-thiopyrano[4,3-*d*]pyrimidine with the thieno[3,2-*d*]pyrimidine structure, resulting in target compounds 4a–d. Similarly, compounds 7a–d were designed and synthesized *via* bioisosterism from compounds 4a–d. To our disappointment, compounds 4a–d and 7a–d didn't exhibit excellent antiproliferative activity as expected against the tested cancer cell lines. To our delight, compounds 7a–d showed better activity than compounds 4a–d. Therefore, we continued to structurally modify the compounds based on the series of compounds 7a–d. Inspired by anti-NSCLC inhibitor AZD-9291, we replaced the morpholine by *N*,*N*,*N*-trimethylethylenediamine and reintroduced the chiral carbon and aryl group to the target compounds. As a result, compounds 13a–l were designed and synthesized. The design strategy for all target compounds is shown in Fig. 2.

PI3K–Akt–mTOR pathway plays a key role in the growth and proliferation of many kinds of cancer such as lung cancer, breast cancer, prostate cancer, lymphoma, ovarian cancer and liver cancer.^10,11^ It was reported that the A549 (human lung cancer), HepG2 (human liver cancer), and MCF-7 (human breast cancer) cancer cell lines are highly sensitive to PI3K/Akt/mTOR signaling pathway inhibitors.^12^ Therefore, we disclosed the synthesis and antiproliferative activity of all the target compounds against the A549, HepG2, and MCF-7 cancer cell lines, and the inhibitory activity against PI3Kα and mTOR kinases. Moreover, time- and dose-dependent experiments, AO and Annexin V-FITC/PI staining and docking studies were also carried out.

## 2. Chemistry

**Scheme 1:**
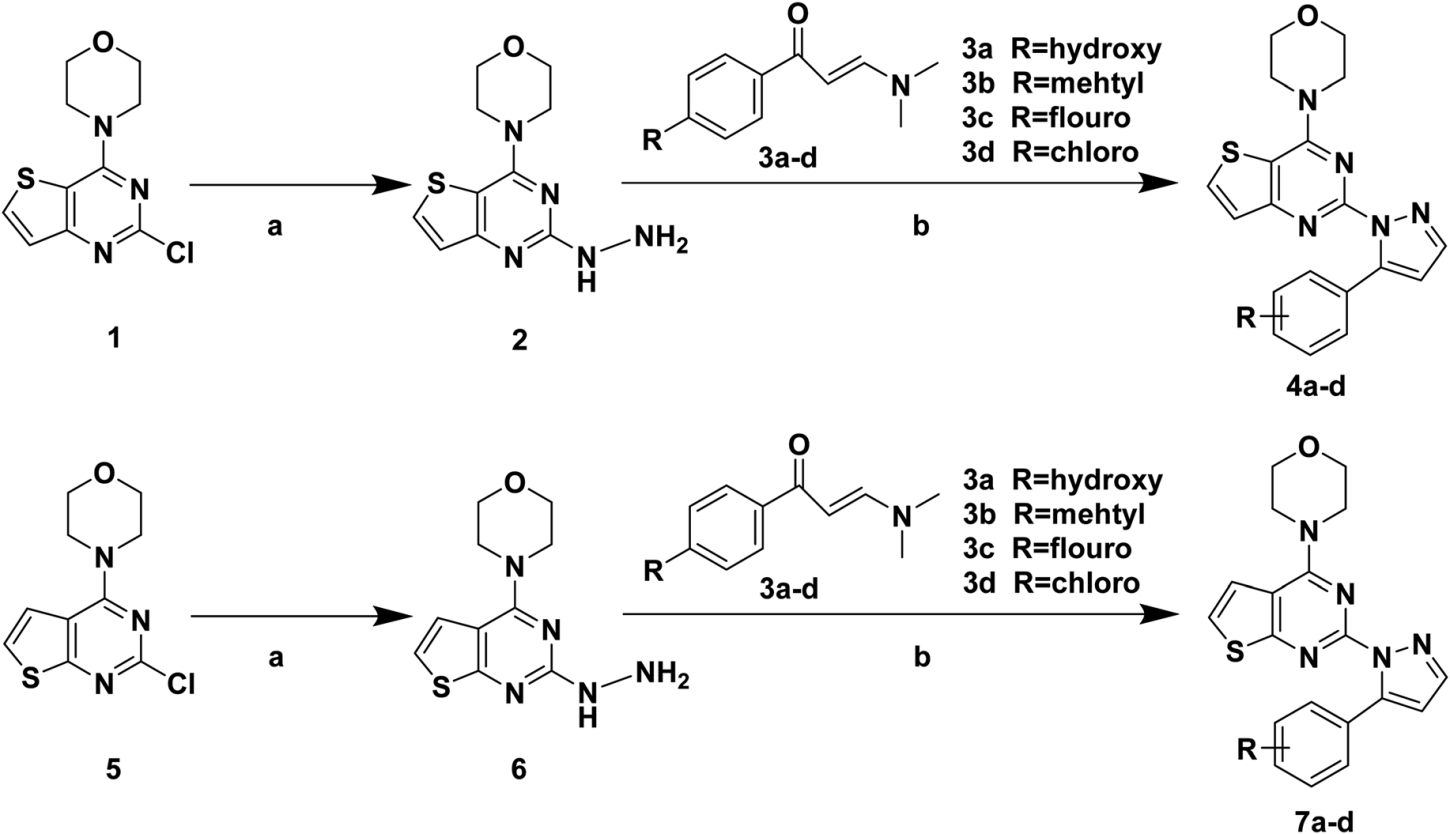
Synthetic routes of target compounds. Reagents and conditions: (a) 80% NH_2_NH_2_·H_2_O, EtOH, 78 °C, 1 h; (b) 1 eq. HCl, EtOH, 70 °C, 6 h.

**Scheme 2:**
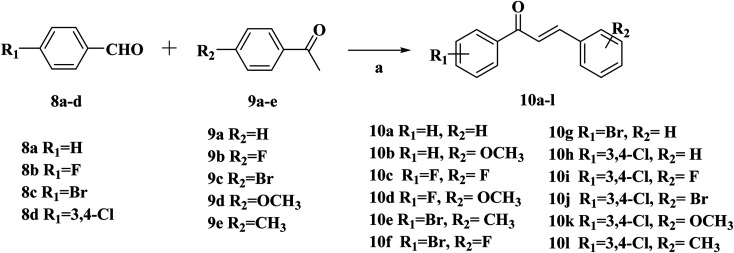
Synthetic routes of chalcone 10a–l. Reagents and conditions: (a) 10% NaOH, EtOH, r.t., 24 h.

**Scheme 3:**
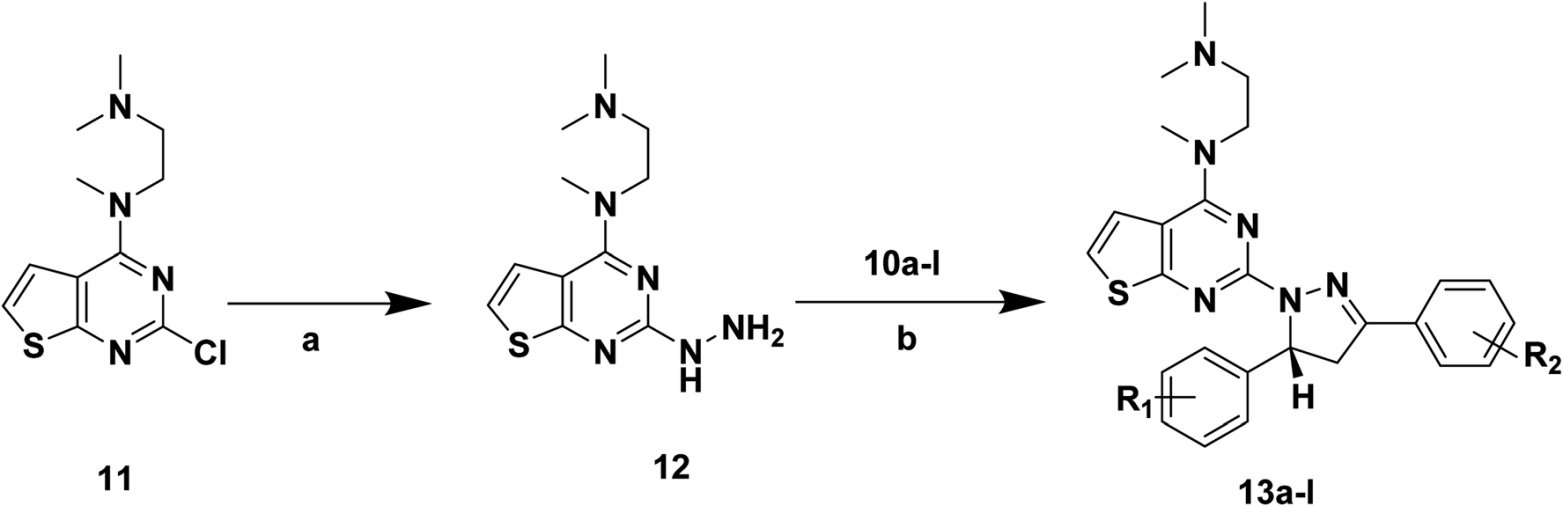
Synthetic routes of target compounds. Reagents and conditions: (a) 80% NH_2_NH_2_·H_2_O, EtOH, 78 °C, 1 h; (b) glacial acetic acid, H_2_SO_4_; 100 °C.

The synthetic routes of target compounds 4a–d, 7a–d and 13a–l are described in Schemes 1–3.

The intermediates (1, 5 and 11) were synthesized from commercially available methyl 2-aminothiophene-3-carboxylate or ethyl 2-amino-4,5,6,7-tetrahydrobenzo[*b*]thiophene-3-carboxylate through five steps or six steps, respectively, which were reported in our previously research.^10^ Then, intermediates 1, 5 and 11 were each treated with 80% hydrazine monohydrate in refluxing ethanol, generating 2, 6 and 12, respectively.^13,14^ Nextly, 2 and 6 were each reacted with phenylpropenone (3a–d), and the reaction was carried out for 1 hour to obtain the compounds 4a–d and 7a–d. The key intermediate 12 condensed with the corresponding chalcone (10a–l) to afford the target compounds 13a–l.

## 3. Results and discussion

### 3.1. Biological evaluation

Taking GDC-0941 as the reference compound, the target compounds (4a–d, 7a–d and 13a–l) were evaluated for the antiproliferative activity against three cancer cell lines A549, MCF-7, and HepG2 by the 3-(4,5-dimethyl-2-thiazolyl)-2,5-diphenyl-2-*H*-tetrazolium bromide (MTT) method. In addition, the activity against PI3Kα/mTOR kinase of the most promising compound 13f was further evaluated. The results expressed as IC_50_ values were summarized in Tables 1–3, where the values were the average of at least two independent experiments.

**Table 1:** *In vitro* cell viability of target compound 4a–d, 7a–d

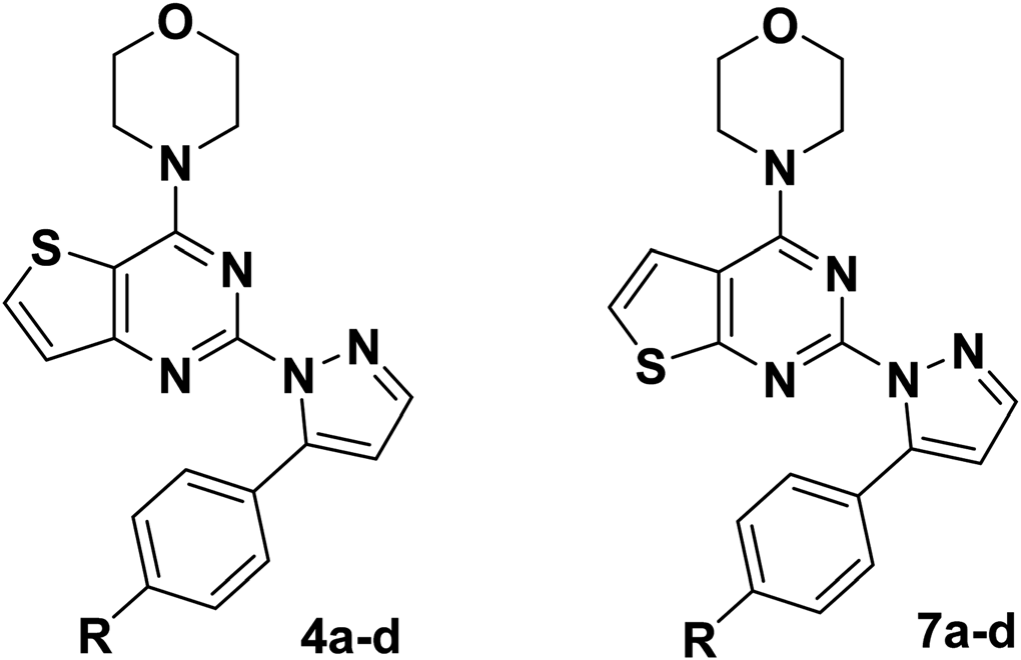				
Compd	*R*	IC_50_^a^ (μM)		
		A549	MCF-7	HePG2
4a	4-H	>100	>100	>100
4b	4-CH_3_	>100	>100	>100
4c	4-F	>100	>100	>100
4d	4-Cl	>100	>100	>100
7a	4-H	37.62 ± 1.88	25.30 ± 0.93	20.16 ± 1.15
7b	4-CH_3_	35.04 ± 0.97	30.95 ± 1.05	33.94 ± 1.13
7c	4-F	>100	>100	>100
7d	4-Cl	>100	>100	>100
GDC-0941^b^	—	6.99 ± 0.21	0.07 ± 0.03	0.20 ± 0.08

a The values are an average of two separate determinations.

b Used as a positive controls.

From the results in Table 1, most of the compounds of 4a–d and 7a–d were inactive against the three cancer cells. Among them, compounds 7a and 7b showed better activity than the others against the three tested cancer cell lines, and the antiproliferative activity of these two compounds against MCF-7 and HepG2 cancer cell lines was better than that of A549 cancer cell lines. In the series of compound 7a–d, the substituent *R* on the benzene ring has an effect on the antiproliferative activity of the compound, and when substituent *R* is hydrogen and the methyl group, it's contributes an increase to the antiproliferative activity of the compound. Taken compounds 7a–d and 4a–d as comparison, we found that compounds with thieno[2,3-*d*]pyrimidine as the backbone structure showed better antiproliferative activity than the compounds with thieno[3,2-*d*]pyrimidine as the backbone structure.

From the results in Table 2, most of the compounds showed moderate to excellent antiproliferative activity against three cancer cells lines (A549, HepG2 and MCF-7). Among them, three compounds (13f, 13g and 13h) showed better activity against A549 cells lines compared with the positive compound GDC-0941. The most promising compound 13f exhibited the best activity against A549, HepG2 and MCF-7 cancer cell lines with the IC_50_ values of 2.84 ± 0.09 μM, 2.08 ± 0.36 μM and 2.88 ± 0.43 μM, respectively. The antiproliferative activity of compound 13f against A549 cancer cell lines was comparable to the positive control GDC-0941 (6.99 ± 0.21 μM). On the whole, the morpholine is replaced by *N*,*N*,*N*-trimethylethylenediamine, and the chiral carbon and aryl groups are reintroduced into the target compound, which is advantageous for the antiproliferative activity of the compound. The introduction of substituents on *R*_1_ and *R*_2_ has different effects on antiproliferative activity, and the introduction of electron donating groups is more conducive to improve antiproliferative activity. The effect on the activity about the introduction of substituents is probably related to a change in the electronic configuration of the benzopyrazole structure. What's more, compounds substituted with electron donating groups (CH_3_, –OCH_3_) in *R*_1_ group showed better activity than those with none substituent. In general, it's seemed to be that target compounds with electron donating groups (EDGs) have a significant impact on the *in vitro* activity, such as compounds 13c, 13f, 13g and 13h.

**Table 2:** *In vitro* cell viability of target compound 13a–l

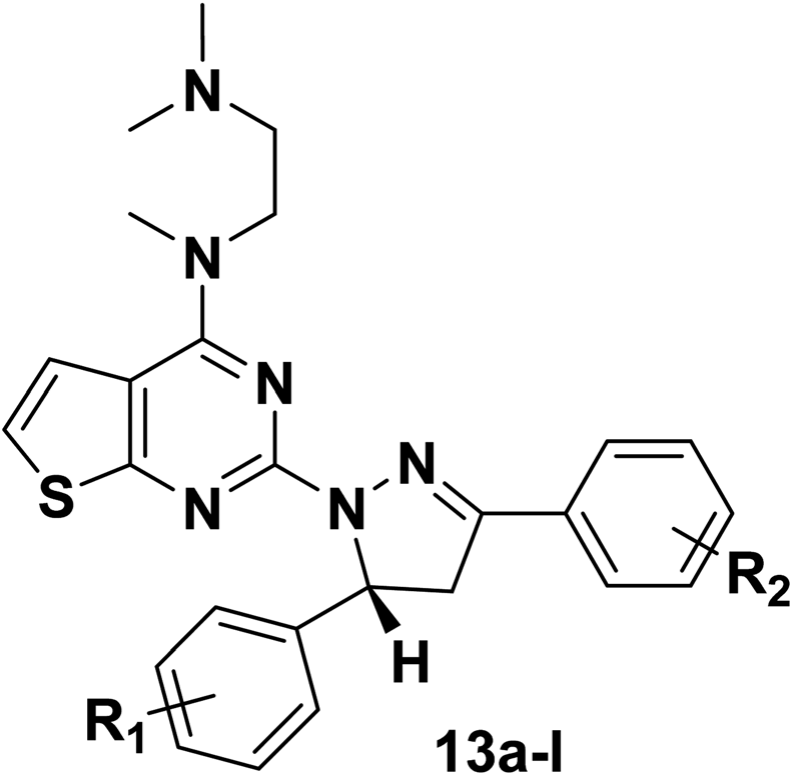					
Compd	*R*_1_	*R*_2_	IC_50_^a^ (μM)		
			A549	MCF-7	HepG2
13a	4-OCH_3_	3,4-Di Cl	NA	NA	NA
13b	4-Br	3,4-Di Cl	17.43 ± 0.13	12.82 ± 0.23	12.90 ± 0.64
13c	4-F	4-F	9.23 ± 0.02	6.51 ± 0.31	6.66 ± 0.13
13d	4-F	3,4-Di Cl	9.17 ± 0.32	6.82 ± 0.17	7.27 ± 0.68
13e	4-H	3,4-Di Cl	13.41 ± 0.29	10.48 ± 0.54	8.05 ± 0.05
13f	4-OCH_3_	4-H	2.84 ± 0.09	2.88 ± 0.43	2.08 ± 0.36
13g	4-CH_3_	3,4-Di Cl	8.75 ± 0.24	4.98 ± 0.04	6.96 ± 0.44
13h	4-CH_3_	4-Br	5.43 ± 0.17	NA	4.45 ± 0.25
13i	4-H	4-H	9.43 ± 0.10	5.04 ± 0.06	6.70 ± 0.14
13j	4-OCH_3_	4-F	NA	NA	83.86 ± 0.17
13k	4-F	4-Br	22.65 ± 0.35	18.39 ± 0.18	10.89 ± 0.20
13l	4-H	4-Br	22.95 ± 0.15	17.17 ± 0.48	15.79 ± 1.23
GDC-0941^b^	—	—	6.99 ± 0.21	0.20 ± 0.08	0.07 ± 0.03

a The values are an average of two separate determinations.

b Used as a positive controls.

Compared 4a–d, 7a–d and 13a–l, it was found that the introduction of *N*,*N*,*N*-trimethylethylenediamine and aryl group to the thieno[2,3-*d*]pyrimidine part and reintroduced the chiral carbon had a significant impact to the antiproliferative activity. The electron cloud density distribution in the molecule of the compound is adapted to the specific site of the protein receptor, which facilitates the binding of the compound to the receptor and enhances the activity of the compounds. The 5-position benzene ring structure and *N*,*N*,*N*-trimethylethylenediamine structure may promote the tight binding of the compounds to the protein receptor.

Activity against PI3Kα and mTOR kinase of four selected compounds 13c, 13f, 13g and 13h was further carried out in this paper. As shown in Table 3, these four selected compounds have better inhibitory activity with IC_50_ values ranging from 0.92 μM to 7.16 μM against PI3Kα kinase than that against mTOR kinase with IC_50_ higher than 10 μM. Overall, the inhibitory activity of the above four compounds against PI3Kα kinase is much better than that of mTOR kinase and compound 13f is the most potential selective PI3Kα inhibitor.

**Table 3:** PI3Kα/mTOR kinase activity and cytotoxicity of selected compounds and positive controls

Compd	IC_50_^a^ (μM)	
	PI3Kα^c^	mTOR^d^
13c	4.62 ± 0.54	>10
13f	0.92 ± 0.14	>10
13g	3.31 ± 0.12	>10
13h	7.16 ± 0.27	>10
GDC-0941^b^	0.003	0.58

a The values are an average of two separate determinations.

b Used as a positive controls.

c PI3Kα: phosphatidylinositol-3-kinase alpha subunit.

d mTOR: mammalian target of rapamycin.

### 3.2. Dose-dependent and time-dependent *in vitro* effects

**Fig. 3:**
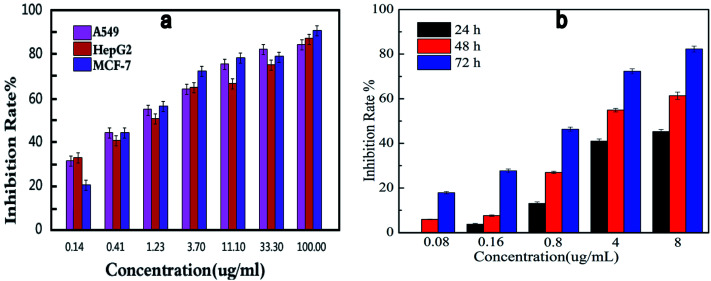
Dose-dependent (a) and time-dependent (b) *in vitro* effects of compound 13f on inhibitory activity against A549.

Comparing the three series of compounds, the compound 13f showed the best antiproliferative activity and enzyme inhibitory activity. To further confirm whether the concentration of this compound has an effect on the inhibition of cell viability, the compound 13f was formulated into seven different concentrations, and the inhibition rate of the seven concentrations on the three cancer cells was measured by the MTT method. As shown in Fig. 3a, the higher the concentration of the compound was, the better the inhibition activity was. The inhibition rate was positively correlated with the compound concentration, and the inhibition to cancer cells was dose-dependent.

In order to investigate the effect of time on inhibition of cell viability, compound 13f was formulated into five different concentration gradients, and the inhibition rate of the compound against the same concentration of A549 cancer cell lines was determined by MTT assay. As shown in Fig. 3b, at the same concentration, the inhibition rate of compound 13f on A549 cancer cell lines was positively correlated with time, the longer the compound 13f was exposed to A549 cancer cell lines, the higher the inhibition rate was.

Summarizing the relationship between the dose, time and the inhibition rate of compound 13f on A549 cancer cell lines, it was found that compound 13f inhibited the proliferation of A549 cancer cell lines in a dose-dependent and time-dependent manner.

### 3.3. Morphology of cells by fluorescence microscopy

**Fig. 4:**
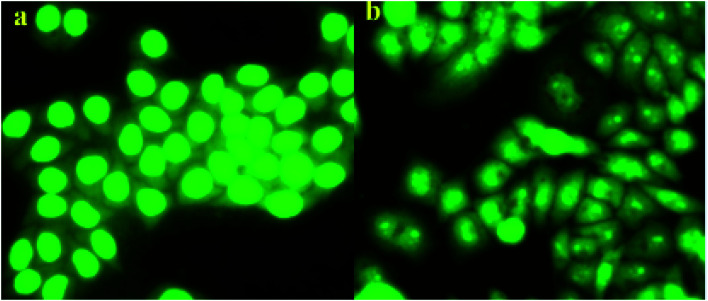
Effect of compound 13f on A549 cell by acridine orange (AO) single staining. ((a) is the control group, (b) is the test group).

In order to investigate whether the target compound 13f could induce the apoptosis of A549 cancer cell lines, acridine orange (AO) staining assay was carried out. As show in the Fig. 4, the control group cell (Fig. 4a) was treated with nothing and showed normal A549 cell shape. But in the test group (Fig. 4b), the cell shape was abnormal with cell shrinkage, chromatin condensation after compound 13f acted on the A549 cells. The results indicated that the compounds 13f could induce apoptosis of A549 cancer cell lines.

### 3.4. Apoptosis result analyzing

**Fig. 5:**
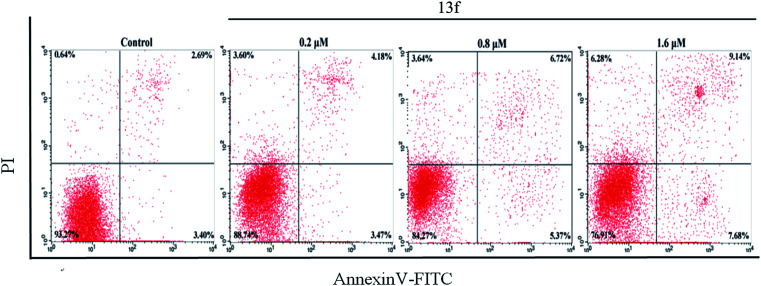
Effect of compound 13f induced apoptosis on A549 cells using Annexin V-FITC and propidium iodide (PI) double staining by flow cytometry.

In order to further reveal the mechanism of apoptosis of A549 induced by the compound 13f, the experiment of Annexin V-FITC and propidium iodide (PI) double staining was carried out. The results were shown in Fig. 5. Compared with the control group, compound 13f with concentration of 1.6 μM could induce the late apoptotic significantly, with 7.68% late apoptotic and 9.14% early apoptotic. And the same trend was also observed in other concentration. In addition, the number of apoptotic and dead cells increased with increasing concentration of the compound. From this, it was found that the target compound 13f can induce apoptosis in a concentration-dependent manner.

Through in-depth research, it was found that the synthesized target compounds can inhibit the proliferation of A549 cells in a time-dependent and dose-dependent manner. AO staining and Annexin V-FITC/PI flow cytometry showed the compound 13f could induce apoptosis of human lung cancer A549 cells.

### 3.5. Molecular docking study

**Fig. 6:**
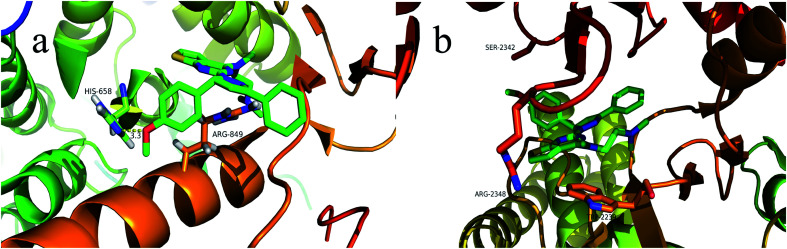
Binding modes of compound 13f with PI3Kα/mTOR (a show the binding pattern of 13f to the PI3Kα protein kinase; b shows the pattern binding of 13f to the mTOR protein kinase).

To explore the binding modes of target compounds with the active site of PI3Kα/mTOR, molecular docking simulation studies were carried out by the AutoDock4.2 software and the docking results were processed and modified in PyMOL 1.8.x software. Based on the *in vitro* inhibition results, we selected compound 13f as ligand example, the structures of PI3Kα (PDB ID code: 3BDS) and mTOR (PDB ID code: 4JSV) were selected as the docking models. The results were shown in Fig. 6.

In the docking model of compound 13f and PI3Kα protein kinase (Fig. 6a), the 5-position benzene ring structure is tightly packed by the hydrophobic group and is in a hydrophobic cavity, allowing the compound to bind tightly to the protein receptor. The *N*,*N*,*N*-trimethylethylenediamine structure forms a hydrogen bond with the hydrophilic amino acid residue HIS-658, which moderately enhances the solubility of the compound. Pyrazoline and pyrimidine ring structure are close to the hydrophobic amino acid residue ARG-849. As shown in Fig. 6b, it can be observed that compound 13f has no binding to any amino acid residues in the periphery. This further explains the reason why compound 13f is less active against mTOR kinase. These results of the molecular docking study showed that compound 13f may be a potential PI3Kα inhibitor.

## 4. Conclusions

In summary, a series of thienopyrimidines containing pyrazoline unit (4a–d, 7a–d and 13a–l) were designed and synthesized. The pharmacological results indicated that most of the compounds showed moderate antiproliferative activity against three cancer cell lines. In particular, the compound 13f showed the best activity against A549, MCF-7 and HepG2 cancer cell lines, with the IC_50_ values of 2.84 ± 0.09 μM, 2.88 ± 0.43 μM and 2.08 ± 0.36 μM, respectively. Four selected compounds (13c, 13f, 13g and 13h) were further evaluated for the inhibitory activity against PI3Kα/mTOR protein kinase. In addition, based on further studies on the compound 13f, it was found that 13f can inhibit the proliferation of A549 cancer cell lines in a time-dependent and dose-dependent manner. AO staining and Annexin V-FITC/PI flow cytometry showed that the compound 13f induced apoptosis of A549 cancer cell lines. The results prompted us that compound 13f may be a potential selective PI3Kα inhibitor.

## 5. Experimental section

### 5.1. Chemistry

All melting points were obtained on a Büchi Melting Point B-540 apparatus (Büchi Labortechnik, Flawil, Switzerland) and were uncorrected. NMR spectra were performed using Bruker 400 MHz spectrometers (Bruker Bioscience, Billerica, MA, USA) with TMS as an internal standard. Mass spectra (MS) were taken in ESI mode on Agilent 1100LCMS (Agilent, Palo Alto, CA, USA). TLC analysis was carried out on silica gel plates GF254 (Qingdao Haiyang Chemical, China). All the materials were obtained from commercial suppliers and used without purification, unless otherwise specified. Yields were not optimized.

### 5.2. General procedure for the preparation of compounds 1, 5 and 11

Compounds 1, 5 and 11 were synthesized according to the reported procedures by our research group.^13,14^

### 5.3. General procedure for the preparation of compounds 2, 6 and 12

Dissolve 2.5 g of 1 (4-(2-chlorothieno[2,3-*d*]pyrimidin-4-yl)morpholine) or 5 (4-(2-chlorothieno[3,2-*d*] pyrimidin-4-yl)morpholine) in 100 mL of hydrazine hydrate. Reaction at 80 °C for 5 hours, cool down to room temperature and precipitate a pale yellow solid. The solid was filtered off and the filter cake was dried to give the product.^15^

### 5.4. General procedure for the preparation of target compounds 4a–d and 7a–d

2 (4-(2-Mercaptothieno[2,3-*d*]pyrimidin-4-yl)morpholine) or 6 (4-(2-mercaptothieno[3,2-*d*]pyrimidin-4-yl)morpholine) (0.001 mol) and 3a–d (phenyl propyl ketone) (0.001 mol) were dissolved in ethanol (12 mL), 1 eq. of HCl was used as a catalyst, and reacted at 70 °C for 6 hours. The reaction was stopped, cooled to room temperature, and the reaction solution was adjusted to a pH of 6–7. Solid precipitated, suction filtered, and the filter cake dried. Due to the formation of by-products in the reaction, the dried filter cake was separated by column chromatography with CH_2_Cl_2_ : AcOH = 4 : 1, and the solvent was removed under reduced pressure to obtain pure target product 4a–d or 7a–d.

#### 4-(2-(5-Phenyl-1*H*-pyrazol-1-yl)thieno[3,2-*d*]pyrimidin-4-yl)morpholine (4a)

A yellow solid, mp 243.6–244.8 °C; ESI-MS *m*/*z*: [M + H]^+^: 363.4; ^1^H-NMR (400 MHz, DMSO) *δ* 8.73 (d, *J* = 2.5 Hz, 1H), 7.95 (d, *J* = 7.4 Hz, 2H), 7.69 (d, *J* = 6.2 Hz, 1H), 7.60 (s, 1H), 7.46 (s, 2H), 7.37 (t, *J* = 7.3 Hz, 1H), 7.06 (s, 1H), 4.04–3.95 (m, 4H), 3.81–3.73 (m, 4H).

#### 4-(2-(5-(*p*-Tolyl)-1*H*-pyrazol-1-yl)thieno[3,2-*d*]pyrimidin-4-yl)morpholine (4b)

A yellow solid, mp 166.8–171.5 °C; ESI-MS *m*/*z*: [M + H]^+^: 377.1; ^1^H-NMR (400 MHz, DMSO) *δ* 8.71 (s, 1H), 7.84 (d, *J* = 7.8 Hz, 2H), 7.68 (d, *J* = 6.1 Hz, 1H), 7.59 (d, *J* = 6.1 Hz, 1H), 7.27 (d, *J* = 7.7 Hz, 2H), 7.00 (s, 1H), 3.98 (d, *J* = 4.2 Hz, 4H), 3.77 (s, 4H), 2.34 (s, 3H).

#### 4-(2-(5-(4-Fluorophenyl)-1*H*-pyrazol-1-yl)thieno[3,2-*d*]pyrimidin-4-yl)morpholine (4c)

A yellow solid, mp 228–231 °C; ESI-MS *m*/*z*: [M + H]^+^: 381.4; ^1^H-NMR (400 MHz, DMSO) *δ* 8.73 (d, *J* = 2.5 Hz, 1H), 8.00 (dd, *J* = 8.4, 5.6 Hz, 2H), 7.69 (d, *J* = 6.1 Hz, 1H), 7.59 (d, *J* = 6.1 Hz, 1H), 7.29 (t, *J* = 8.8 Hz, 2H), 7.05 (d, *J* = 2.5 Hz, 1H), 3.99 (d, *J* = 4.4 Hz, 4H), 3.78 (d, *J* = 4.3 Hz, 4H).

#### 4-(2-(5-(4-Chlorophenyl)-1*H*-pyrazol-1-yl)thieno[3,2-*d*]pyrimidin-4-yl)morpholine (4d)

A yellow solid, mp 250–251 °C; ESI-MS *m*/*z*: [M + H]^+^: 397.8; ^1^H-NMR (400 MHz, DMSO) *δ* 8.74 (d, *J* = 2.5 Hz, 1H), 7.98 (d, *J* = 8.4 Hz, 2H), 7.69 (d, *J* = 6.1 Hz, 1H), 7.60 (d, *J* = 6.1 Hz, 1H), 7.52 (d, *J* = 8.4 Hz, 2H), 7.08 (d, *J* = 2.5 Hz, 1H), 3.99 (s, 4H), 3.78 (d, *J* = 4.3 Hz, 4H).

#### 4-(2-(5-Phenyl-1*H*-pyrazol-1-yl)thieno[2,3-*d*]pyrimidin-4-yl)morpholine (7a)

An orange yellow solid, mp 223–225 °C; ESI-MS *m*/*z*: [M + H]^+^: 363.4; ^1^H-NMR (400 MHz, DMSO) *δ* 8.75 (s, 1H), 8.31 (dd, *J* = 5.5, 1.9 Hz, 1H), 7.96 (d, *J* = 7.2 Hz, 2H), 7.54 (dd, *J* = 5.5, 1.9 Hz, 1H), 7.47 (q, *J* = 7.4 Hz, 2H), 7.37 (t, *J* = 7.8 Hz, 1H), 7.08–7.00 (m, 1H), 4.03 (s, 4H), 3.83–3.77 (m, 4H).

#### 4-(2-(5-(*p*-Tolyl)-1*H*-pyrazol-1-yl)thieno[2,3-*d*]pyrimidin-4-yl)morpholine (7b)

A light yellow solid, mp 286–289 °C; ESI-MS *m*/*z*: [M + H]^+^: 377.4; ^1^H-NMR (400 MHz, DMSO) *δ* 8.82 (d, *J* = 2.1 Hz, 1H), 8.41 (d, *J* = 5.5 Hz, 1H), 7.95 (d, *J* = 7.9 Hz, 2H), 7.64 (d, *J* = 5.4 Hz, 1H), 7.37 (d, *J* = 7.9 Hz, 2H), 7.10 (d, *J* = 2.2 Hz, 1H), 4.13 (s, 4H), 3.91 (d, *J* = 4.2 Hz, 4H), 2.45 (s, 3H).

#### 4-(2-(5-(4-Fluorophenyl)-1*H*-pyrazol-1-yl)thieno[2,3-*d*]pyrimidin-4-yl)morpholine (7c)

A light yellow solid, mp 202–205 °C; ESI-MS *m*/*z*: [M + H]^+^: 381.4; ^1^H-NMR (400 MHz, DMSO) *δ* 8.85 (d, *J* = 2.6 Hz, 1H), 8.46–8.39 (m, 1H), 8.15–8.08 (m, 2H), 7.66–7.62 (m, 1H), 7.40 (t, *J* = 8.9 Hz, 2H), 7.14 (d, *J* = 2.6 Hz, 1H), 4.13 (s, 4H), 3.91 (d, *J* = 4.5 Hz, 4H).

#### 4-(2-(5-(4-Chlorophenyl)-1*H*-pyrazol-1-yl)thieno[2,3-*d*]pyrimidin-4-yl)morpholine (7d)

A yellow solid, mp 280–282 °C; ESI-MS *m*/*z*: [M + H]^+^: 397.8; ^1^H-NMR (400 MHz, DMSO) *δ* 8.63 (d, *J* = 2.6 Hz, 1H), 8.19 (d, *J* = 5.5 Hz, 1H), 7.86 (d, *J* = 8.5 Hz, 2H), 7.41 (dd, *J* = 6.9, 4.8 Hz, 3H), 6.95 (d, *J* = 2.7 Hz, 1H), 3.95–3.84 (m, 4H), 3.72–3.62 (m, 4H).

### 5.5. General procedure for the preparation of target compounds 10a–l

Compounds 10a–l were synthesized according to the reported procedures by our research group.^13^

### 5.6. General procedure for the preparation of target compounds 13a–l

10a–l (chalcone) (0.001 mol) and 12 (*N*^1^-(2-mercaptothieno[2,3-*d*]pyrimidin-4-yl)-*N*^1^,*N*^2^,*N*^2^-trimethylethane-1,2-diamine) (0.001 mol) dissolved in 20 mL of acetic acid, concentrated H_2_SO_4_ is the catalyst. The reaction solution was heated on a 100–110 °C oil bath, and the reaction was confirmed by TLC, and excess solvent was removed under reduced pressure. The precipitated solid is suction filtered, washed with ethanol, dried, and recrystallize with a suitable solvent mixture. The target compound 13a–1 is obtained.

#### *N* ^1^-(2-(3-(3,4-Dichlorophenyl)-5-(4-methoxyphenyl)-4,5-dihydro-1*H*-pyrazol-1-yl)thieno[2,3-*d*]pyrimidin-4-yl)-*N*^1^,*N*^2^,*N*^2^-trimethylethane-1,2-diamine (13a)

A white solid, mp 232–233 °C; ESI-MS *m*/*z*: [M + H]^+^: 555.2; ^1^H-NMR (400 MHz, DMSO) *δ* 8.14 (d, *J* = 5.5 Hz, 1H), 7.76 (d, *J* = 8.5 Hz, 2H), 7.68–7.54 (m, 2H), 7.33–7.18 (m, 2H), 7.07 (d, *J* = 8.7 Hz, 2H), 6.02–5.74 (m, 1H), 4.03 (d, *J* = 4.5 Hz, 2H), 3.89 (s, 1H), 3.86 (s, 3H), 3.62 (s, 1H), 3.46 (s, 3H), 3.28–3.17 (m, 2H), 2.84 (s, 6H).

#### *N* ^1^-(2-(5-(4-Bromophenyl)-3-(3,4-dichlorophenyl)-4,5-dihydro-1*H*-pyrazol-1-yl)thieno[2,3-*d*]pyrimidin-4-yl)-*N*^1^,*N*^2^,*N*^2^-trimethylethane-1,2-diamine (13b)

A white solid, mp 291–294 °C; ESI-MS *m*/*z*: [M + H]^+^: 604.3; ^1^H-NMR (400 MHz, DMSO) *δ* 8.05 (d, *J* = 5.4 Hz, 1H), 7.69 (d, *J* = 8.6 Hz, 2H), 7.64 (d, *J* = 8.5 Hz, 2H), 7.55 (d, *J* = 8.2 Hz, 1H), 7.51 (s, 1H), 7.25–7.08 (m, 2H), 5.79 (dd, *J* = 12.0, 5.0 Hz, 1H), 3.86 (dd, *J* = 17.7, 12.3 Hz, 2H), 3.63 (s, 2H), 3.48 (s, 1H), 3.15 (dd, *J* = 17.5, 5.4 Hz, 2H), 2.57 (d, *J* = 2.6 Hz, 2H), 2.37 (s, 6H).

#### *N* ^1^-(2-(3,5-Bis(4-fluorophenyl)-4,5-dihydro-1*H*-pyrazol-1-yl)thieno[2,3-*d*]pyrimidin-4-yl)-*N*^1^,*N*^2^,*N*^2^-tri-methylethane-1,2-diamine (13c)

A white solid, mp 256–257 °C; ESI-MS *m*/*z*: [M + H]^+^: 492.5; ^1^H-NMR (400 MHz, DMSO) *δ* 8.03 (d, *J* = 5.5 Hz, 1H), 7.84–7.75 (m, 2H), 7.48 (d, *J* = 8.2 Hz, 2H), 7.28 (t, *J* = 8.6 Hz, 2H), 7.18 (t, *J* = 7.7 Hz, 3H), 5.75 (dd, *J* = 12.3, 4.4 Hz, 1H), 3.86 (dd, *J* = 17.6, 12.1 Hz, 1H), 3.73 (d, *J* = 7.0 Hz, 1H), 3.55 (d, *J* = 13.6 Hz, 1H), 3.41 (s, 2H), 3.17 (s, 1H), 3.08 (dd, *J* = 17.5, 4.7 Hz, 1H), 2.36 (s, 2H), 2.20 (s, 6H).

#### *N* ^1^-(2-(3-(3,4-Dichlorophenyl)-5-(4-fluorophenyl)-4,5-dihydro-1*H*-pyrazol-1-yl)thieno[2,3-*d*]pyrimidin-4-yl)-*N*^1^,*N*^2^,*N*^2^-trimethylethane-1,2-diamine (13d)

A white solid, mp 244–247 °C; ESI-MS *m*/*z*: [M + H]^+^: 543.4; ^1^H-NMR (400 MHz, DMSO) *δ* 8.06 (d, *J* = 5.5 Hz, 1H), 7.83–7.77 (m, 2H), 7.58–7.51 (m, 2H), 7.29 (t, *J* = 8.8 Hz, 2H), 7.19 (dd, *J* = 14.5, 6.9 Hz, 2H), 5.80 (dd, *J* = 12.4, 5.0 Hz, 1H), 3.88 (dd, *J* = 17.5, 12.4 Hz, 2H), 3.46 (s, 2H), 3.18 (dd, *J* = 17.7, 5.1 Hz, 3H), 2.83 (s, 2H), 2.56 (s, 6H).

#### *N* ^1^-(2-(3-(3,4-Dichlorophenyl)-3-phenyl-4,5-dihydro-1*H*-pyrazol-1-yl)thieno[2,3-*d*]pyrimidine-4-yl)-*N*^1^, *N*^2^,*N*^2^-trimethylethane-1,2-diamine (13e)

A white solid, mp 262–263 °C; ESI-MS *m*/*z*: [M + H]^+^: 525.5; ^1^H-NMR (400 MHz, DMSO) *δ* 8.05 (d, *J* = 5.4 Hz, 1H), 7.77 (d, *J* = 7.6 Hz, 2H), 7.62–7.50 (m, 2H), 7.48–7.37 (m, 3H), 7.20 (dd, *J* = 20.5, 6.8 Hz, 2H), 5.77 (dd, *J* = 12.2, 5.1 Hz, 1H), 3.88 (dd, *J* = 17.7, 12.4 Hz, 1H), 3.79–3.70 (m, 1H), 3.53–3.43 (m, 1H), 3.26 (s, 3H), 3.14 (dd, *J* = 17.7, 5.0 Hz, 1H), 2.20 (s, 2H), 2.11 (s, 6H).

#### *N* ^1^-(2-(5-(4-Methoxyphenyl)-3-phenyl-4,5-dihydro-1*H*-pyrazol-1-yl)thieno[2,3-*d*]pyrimidin-4-yl)-*N*^1^,*N*^2^,*N*^2^-trimethylethane-1,2-diamine (13f)

A white solid, mp 287–289 °C; ESI-MS *m*/*z*: [M + H]^+^: 486.6; ^1^H-NMR (400 MHz, DMSO) *δ* 8.04 (d, *J* = 5.4 Hz, 1H), 7.70 (d, *J* = 8.3 Hz, 2H), 7.30 (t, *J* = 7.7 Hz, 2H), 7.26–7.19 (m, 3H), 7.16 (d, *J* = 5.6 Hz, 1H), 7.00 (d, *J* = 8.4 Hz, 2H), 5.82–5.71 (m, 1H), 3.92–3.84 (m, 2H), 3.80 (s, 3H), 3.46 (s, 2H), 3.17 (s, 1H), 3.09 (d, *J* = 4.1 Hz, 1H), 3.05 (d, *J* = 4.2 Hz, 1H), 2.85 (s, 2H), 2.56 (s, 6H).

#### *N* ^1^-(2-(3-(3,4-Dichlorophenyl)-5-(*p*-tolyl)-4,5-dihydro-1*H*-pyrazol-1-yl)thieno[2,3-*d*]pyrimidin-4-yl)-*N*^1^,*N*^2^,*N*^2^-trimethylethane-1,2-diamine (13g)

A white solid, mp 269–270 °C; ESI-MS *m*/*z*: [M + H]^+^: 539.5; ^1^H-NMR (400 MHz, DMSO) *δ* 8.02 (d, *J* = 5.4 Hz, 1H), 7.65 (d, *J* = 8.0 Hz, 2H), 7.54 (d, *J* = 8.3 Hz, 1H), 7.49 (s, 1H), 7.23 (dd, *J* = 14.9, 6.7 Hz, 3H), 7.16 (d, *J* = 8.3 Hz, 1H), 5.73 (dd, *J* = 12.1, 5.2 Hz, 1H), 3.84 (dd, *J* = 17.6, 12.2 Hz, 1H), 3.76–3.68 (m, 1H), 3.49–3.39 (m, 2H), 3.24 (s, 3H), 2.33 (s, 3H), 2.17 (s, 2H), 2.08 (s, 6H).

#### *N* ^1^-(2-(3-(4-Bromophenyl)-5-(*p*-tolyl)-4,5-dihydro-1*H*-pyrazol-1-yl)thieno[2,3-*d*]pyrimidin-4-yl)-*N*^1^,*N*^2^,*N*^2^-trimethylethane-1,2-diamine (13h)

A white solid, mp 272–273 °C; ESI-MS *m*/*z*: [M + H]^+^:549.5; ^1^H-NMR (400 MHz, DMSO) *δ* 8.08 (d, *J* = 5.5 Hz, 1H), 7.65 (d, *J* = 8.0 Hz, 2H), 7.50 (d, *J* = 8.3 Hz, 2H), 7.24 (dd, *J* = 17.4, 8.2 Hz, 4H), 7.17 (d, *J* = 5.4 Hz, 1H), 5.83 (dd, *J* = 12.0, 4.5 Hz, 1H), 3.86 (dd, *J* = 17.7, 12.0 Hz, 2H), 3.40 (s, 3H), 3.10 (dd, *J* = 17.5, 4.4 Hz, 2H), 2.89 (s, 2H), 2.74 (d, *J* = 7.9 Hz, 6H), 2.34 (s, 3H).

#### *N* ^1^-(2-(3,5-Diphenyl-4,5-dihydro-1*H*-pyrazol-1-yl)thieno[2,3-*d*]pyrimidin-4-yl)-*N*^1^,*N*^2^,*N*^2^-trimethylethane-1,2-diamine (13i)

A white solid, mp 225–227 °C; ESI-MS *m*/*z*: [M + H]^+^: 456.6; ^1^H-NMR (400 MHz, DMSO) *δ* 8.14 (d, *J* = 5.5 Hz, 1H), 7.86 (d, *J* = 6.9 Hz, 2H), 7.53 (dd, *J* = 15.7, 8.1 Hz, 3H), 7.39 (t, *J* = 7.5 Hz, 2H), 7.30 (dd, *J* = 14.4, 6.4 Hz, 4H), 5.90 (dd, *J* = 11.9, 4.4 Hz, 1H), 3.98 (dd, *J* = 17.6, 12.1 Hz, 2H), 3.41 (s, 3H), 3.18 (dd, *J* = 17.5, 4.6 Hz, 2H), 2.88 (s, 2H), 2.60 (s, 6H).

#### *N* ^1^-(2-(3-(4-Fluorophenyl)-5-(4-methoxyphenyl)-4,5-dihydro-1*H*-pyrazol-1-yl)thieno[2,3-*d*]pyrimidin-4-yl)-*N*^1^,*N*^2^,*N*^2^-trimethylethane-1,2-diamine (13j)

A white solid, mp 286–287 °C; ESI-MS *m*/*z*: [M + H]^+^: 504.2; ^1^H-NMR (400 MHz, DMSO) *δ* 8.08 (d, *J* = 5.2 Hz, 1H), 7.71 (d, *J* = 8.9 Hz, 2H), 7.21–7.09 (m, 4H), 7.01 (d, *J* = 8.8 Hz, 3H), 5.84 (d, *J* = 7.0 Hz, 1H), 3.91 (d, *J* = 17.0 Hz, 2H), 3.81 (s, 3H), 3.73 (s, 2H), 3.17 (s, 1H), 3.10 (d, *J* = 13.5 Hz, 3H), 2.85 (s, 1H), 2.74 (s, 6H).

#### *N* ^1^-(2-(3-(4-Bromophenyl)-5-(4-fluorophenyl)-4,5-dihydro-1*H*-pyrazol-1-yl)thieno[2,3-*d*]pyrimidin-4-yl)-*N*^1^,*N*^2^,*N*^2^-trimethylethane-1,2-diamine (13k)

A white solid, mp 291–292 °C; ESI-MS *m*/*z*: [M + H]^+^: 552.1; ^1^H NMR (400 MHz, DMSO) *δ* 8.03 (d, *J* = 5.5 Hz, 1H), 7.84–7.75 (m, 2H), 7.48 (d, *J* = 8.2 Hz, 2H), 7.28 (t, *J* = 8.6 Hz, 2H), 7.18 (t, *J* = 7.7 Hz, 3H), 5.75 (dd, *J* = 12.3, 4.4 Hz, 1H), 3.86 (dd, *J* = 17.6, 12.1 Hz, 1H), 3.73 (d, *J* = 7.0 Hz, 1H), 3.55 (d, *J* = 13.6 Hz, 1H), 3.41 (s, 2H), 3.17 (s, 1H), 3.08 (dd, *J* = 17.5, 4.7 Hz, 1H), 2.36 (s, 2H), 2.20 (s, 6H).

#### *N* ^1^-(2-(3-(4-Bromophenyl)-5-phenyl-4,5-dihydro-1*H*-pyrazol-1-yl)thieno[2,3-*d*]pyrimidin-4-yl)-*N*^1^,*N*^2^,*N*^2^-trimethylethane-1,2-diamine (13l)

A white solid, mp 261–262 °C; ESI-MS *m*/*z*: [M + H]^+^: 534.1; ^1^H-NMR (400 MHz, DMSO) *δ* 8.06 (d, *J* = 5.5 Hz, 1H), 7.75 (d, *J* = 6.9 Hz, 2H), 7.49 (d, *J* = 8.5 Hz, 2H), 7.47–7.38 (m, 3H), 7.18 (dd, *J* = 13.6, 6.9 Hz, 3H), 5.79 (dd, *J* = 11.9, 4.7 Hz, 1H), 3.88 (dd, *J* = 17.5, 12.1 Hz, 2H), 3.47 (d, *J* = 6.8 Hz, 2H), 3.20–3.04 (m, 3H), 2.93 (s, 2H), 2.63 (s, 6H).

### 5.7. Cytotoxicity assay *in vitro*

The target compounds (4a–d, 7a–d and 13a–l) were evaluated antiproliferative activity against A549 (human lung cancer cell), HepG2 (human hepatocellular carcinoma cell), MCF-7 (human breast cancer) by the standard MTT assay, with GDC-0941 as positive control (all cells were obtained from the Shanghai Institute of Biological Sciences, Chinese Academy of Sciences, Shanghai, China). The experiment was conducted according to the method reported by our research group.^16^

### 5.8. PI3Kα and mTOR kinase assay

Four selected compounds (13c, 13f, 13g and 13h) are tested for their activity against PI3Kα and mTOR using a Kinase-Glo® Luminescent Kinase Assay, with GDC-0941 as positive control. The experiment was conducted according to the method reported by our research group.^17^

### 5.9. Acridine orange single staining

The cancer cell apoptotic of target compound 13f were evaluated with A549 cancer cell lines by acridine orange single staining. The experiment was conducted according to the method reported by our research group.^18^

### 5.10. Cell apoptosis assay by flow cytometry

A549 cells were seeded in a 6-well plate at 1 × 10^5^ cells per well and incubated for 24 h. Then treated with compound 13f for 24 h. Cells were harvested and fixed with ice-cold 70% ethanol at 4 °C for 12 h. Ethanol was removed and the cells were washed with cold PBS. Then cells were incubated in 0.5 mL of PBS containing 1 mg mL^−1^ RNase for 30 min at 37 °C. Then the cells were stained with propidium iodide (PI) in the dark for 30 min. The DNA contents was then measured by flow cytometer.

### 5.11. Docking studies

Molecular docking simulation studies were carried out by the AutoDock4.2 software (The Scripps Research Institute, USA). The docking tutorial we used and the detailed AutoDock basic operational methods can be found at: http://autodock.scripps.edu/faqs-help/tutorial. The protein preparation process of flexible docking mainly includes fixing the exact residues, adding hydrogen atoms, removing irrelevant water molecules, adding charges, *etc.* The potent compounds were selected as ligand examples, and the structures of PI3Kα (PDB ID code: 3BDS) and mTOR (PDB ID code: 4JSV) were selected as the docking models. Only the best-scoring ligand–protein complexes were used for the binding site analyses. All the docking results were processed and modified in Open-Source PyMOL 1.8.x software (https://pymol.org).

## Conflicts of interest

There are no conflicts to declare.

## Supplementary Material

RA-009-C9RA06192D-s001

## References

[cit1] Xu W., Yang Z., Lu N. (2015). Cell Adhes. Migr..

[cit2] Ardito F., Giuliani M., Perrone D., Troiano G., Lo Muzio L. (2017). Int. J. Mol. Med..

[cit3] Hirsch E., Costa C., Ciraolo E. (2007). J. Endocrinol..

[cit4] Aksoy E., Saveanu L., Manoury B. (2018). Front. Immunol..

[cit5] Yang X., Zhang X., Huang M., Song K., Li X., Huang M., Meng L., Zhang J. (2017). Sci. Rep..

[cit6] Xu Y. C., Wang X., Chen Y., Chen S. M., Yang X. Y., Sun Y. M., Geng M. Y., Ding J., Meng L. H. (2017). Theranostics.

[cit7] Yang J., Nie J., Ma X., Wei Y., Peng Y., Wei X. (2019). Mol. Cancer.

[cit8] Zhao W., Qiu Y., Kong D. (2017). Acta Pharm. Sin. B.

[cit9] Wang Q., Li X., Sun C., Zhang B., Zheng P., Zhu W., Xu S. (2017). Molecules.

[cit10] Arcaro A., Guerreiro A. (2007). Curr. Genomics.

[cit11] Suvarna V., Murahari M., Khan T., Chaubey P., Sangave P. (2017). Front. Pharmacol..

[cit12] Ceci C., Lacal P. M., Tentori L., De Martino M. G., Miano R., Graziani G. (2018). Nutrients.

[cit13] Sun C., Chen C., Xu S., Wang J., Zhu Y., Kong D., Tao H., Jin M., Zheng P., Zhu W. (2016). Bioorg. Med. Chem..

[cit14] Zhu W., Chen C., Sun C., Xu S., Wu C., Lei F., Xia H., Tu Q., Zheng P. (2015). Eur. J. Med. Chem..

[cit15] Wang L., Xu S., Chen X., Liu X., Duan Y., Kong D., Zhao D., Zheng P., Tang Q., Zhu W. (2018). Bioorg. Med. Chem..

[cit16] Xiao Z., Lei F., Chen X., Wang X., Cao L., Ye K., Zhu W., Xu S. (2018). Arch. Pharm..

[cit17] OuYang Y., Wang C., Zhao B., Xiong H., Xiao Z., Zhang B., Zheng P., Hu J., Gao Y., Zhang M., Zhu W., Xu S. (2018). New J. Chem..

[cit18] Wang L., Xu S., Chen X., Liu X., Duan Y., Kong D., Zhao D., Zheng P., Tang Q., Zhu W. (2018). Bioorg. Med. Chem..

