# Supplementary material for: Synthesis and bioevaluation of thienopyrimidines bearing a pyrazoline unit as selective PI3Kα inhibitors
Source: RSC Adv. 2019 Sep 18;9(51):29579–89. doi: 10.1039/c9ra06192d (PMC9072010; doi:10.1039/c9ra06192d)

Electronic Supplementary Material (ESI) for RSC Advances.
This journal is © The Royal Society of Chemistry 2019

^{1}H NMR Spectral

13c. ^{1}H NMR (400 MHz). Solvent: DMSO

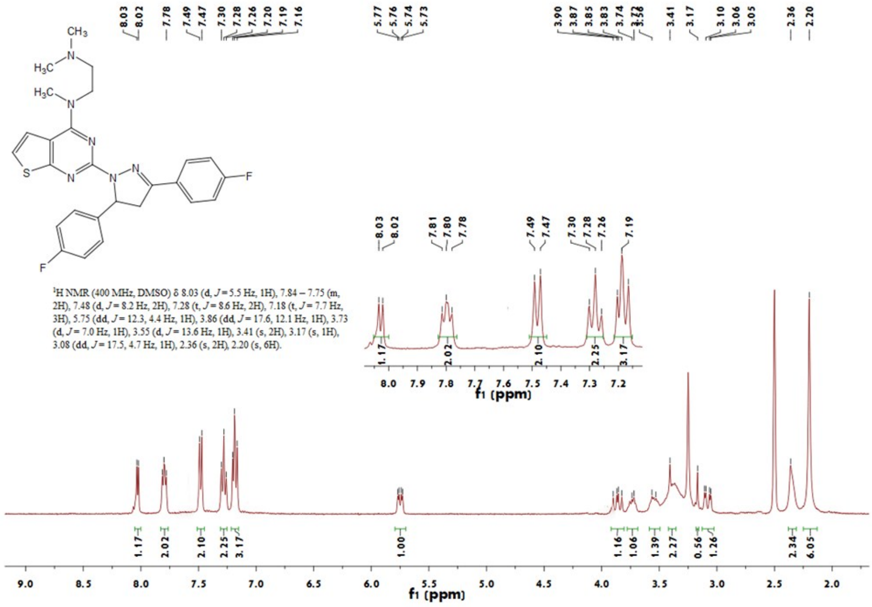

13f. ^{1}H NMR (400 MHz). Solvent: DMSO

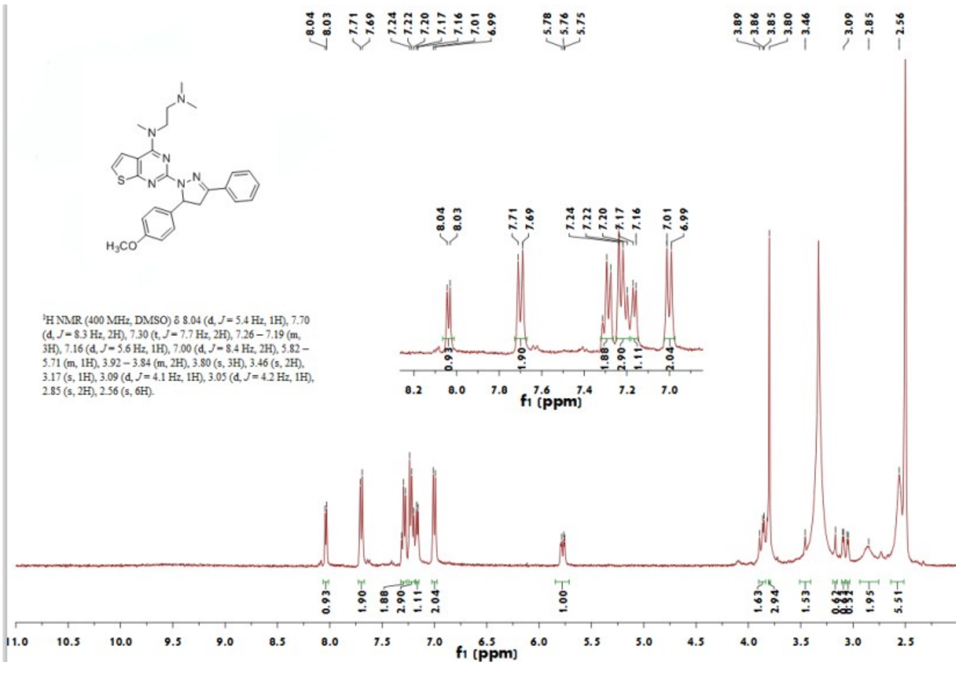

13g. ^{1}H NMR (400 MHz). Solvent: DMSO

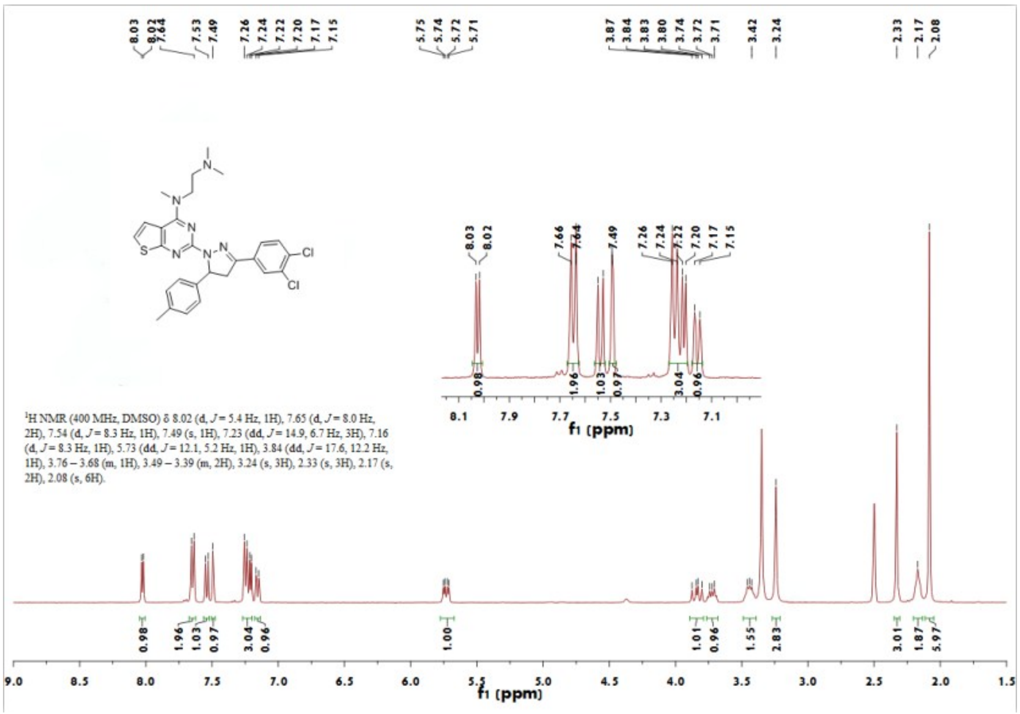

13h. ^{1}H NMR (400 MHz). Solvent: DMSO

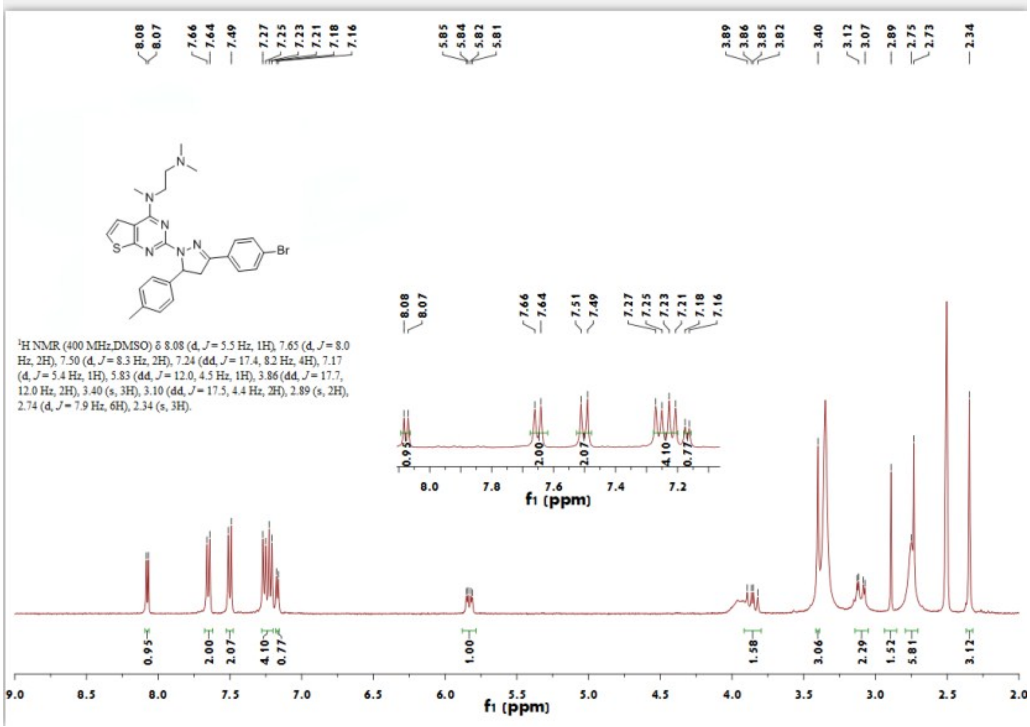

Supplement: RA-009-C9RA06192D-s001 [file RA-009-C9RA06192D-s001.pdf]
